# Recruitment of specific dopamine neuron sub-circuits by opioids

**DOI:** 10.1016/j.addicn.2025.100233

**Published:** 2025-09-23

**Authors:** Sage L. Morison, Luan B. Doster, Andrew D. Vigotsky, Maria V. Centeno, Natalia Lopez Gonzalez del Rey, Apkar V. Apkarian, Rajeshwar Awatramani

**Affiliations:** a The Ken & Ruth Davee Department of Neurology, Northwestern University, Chicago, IL, USA; b UNC Neuroscience Center, The University of North Carolina at Chapel Hill, Chapel Hill, NC, USA; c Department of Cell Biology and Physiology, The University of North Carolina at Chapel Hill, Chapel Hill, NC, USA; d Department of Pharmacology, The University of North Carolina at Chapel Hill, Chapel Hill, NC, USA; e Department of Neuroscience, Northwestern University, Chicago, IL, USA; f Department of Anesthesiology, Northwestern University Feinberg School of Medicine, Chicago, IL, USA; g Department of Physical Medicine & Rehabilitation, Northwestern University Feinberg School of Medicine, Chicago, IL, USA; h Director, Center for Translational Pain Research, Department of Neuroscience, Northwestern University, Chicago, IL, USA; i John Eccles Professor of Neurology, The Ken & Ruth Davee Department of Neurology, Northwestern University, Chicago, IL, USA; j Weinberg College of Arts and Sciences, Northwestern University, Chicago, IL, USA

**Keywords:** Opioids, Dopamine, Heterogeneity, Accumbens, VTA

## Abstract

Opioids have strong addictive and rewarding effects, largely attributed to their interaction with the dopamine (DA) system. Within the ventral tegmental area (VTA), opioids increase DA neural activity by disinhibiting—or decreasing the tonic inhibition of—DA neurons. This disinhibition results in an increase in DA in the nucleus accumbens, which has been widely implicated in addiction. With recent developments suggesting rich VTA DA neuron heterogeneity, a key question is whether opioid exposure activates distinct DA neuron subsets. Here, we address (i) whether this activation is uniformly distributed across the midbrain or, alternatively, is enriched in specific anatomically restricted DA populations, and (ii) the specificity of projections of activated DA neurons, giving insight into the circuitry receiving DA following opioid administration. Using intersectional cFos-based labeling, we find that captured cells are biased towards the VTA over the substantia nigra pars compacta (SNc), specifically towards the *Aldh1a1*-rich paranigral region. We also find that the projection pattern of labeled cells is focused on the dorsomedial shell of the nucleus accumbens compared to the ventromedial shell, core, lateral shell, or dorsal striatum. Projections are also observed in the prefrontal cortex, olfactory tubercle, and basolateral amygdala. Our findings of pathway-specific opioid-induced recruitment of DA neurons provide potential for selective targeting of DA sub-circuits to decrease the addictive nature of opioids without disrupting overall DA function.

## Introduction

Opioids are a class of drugs whose rewarding or addictive effects are considered to be largely dependent on dopamine (DA) release in the nucleus accumbens (ACB) from the ventral tegmental area (VTA) [1,2]. The relationship between DA release and opioids is highly conserved across species [3], considered necessary for associative learning [2,4]. In support of this, DA-receptor deficient animals fail to develop reinforcement to morphine [5,6], a key finding suggesting that opioids’ addictive quality depends on DA release. Furthermore, self-stimulation of VTA DA neurons is on its own reinforcing [7–9], and has been shown to have direct involvement in heroin reinforcement [10]. It is therefore likely that DA release upon opioid exposure is both necessary and sufficient for the rewarding and addictive components of opioids.

The principal target of opioids used in pain management and addictive settings, μ-opioid receptors (MORs) [11], are not robustly detected on most DA neurons. Rather, the DA release effects are principally reliant on disinhibition by neurons that express MORs [1,12,13]. Therefore, opioids primarily exert their effects on upstream inhibitory neurons, which may be located locally in the VTA, in the pars reticulata [14], lateral hypothalamus, rostro-medial tegmental nucleus (RMTg), the periaqueductal gray (PAG), or the striatum [2,15–17]. Indeed, pharmacobehavioral studies indicate that a structure in or very near to the VTA houses the MORs responsible for disinhibition: In rodents, microinjection of morphine into the VTA results in reinforcement behavior [18], MOR-specific blockade in the VTA blunted the effects of heroin and resulted in increased compensatory self-administration [19], while elimination of MOR expression in the midbrain removes its reinforcement altogether [20,21]. Further, locally infused morphine recapitulates the majority of analgesic effects of systemic application [22]. Importantly, it has been suggested that these behavioral effects are mediated by DA release.

However, there is also directly contrasting evidence that questions the existing DA paradigm. When a TH-deficient mouse model was tested, the animals developed VTA-dependent [23] but putatively DA-independent opioid place preference [24,25], bringing into question the necessity of DA while reinforcing the importance of the VTA. Another group added nuance by comparing previously opioid-dependent but withdrawn rats to drug naïve rats and found only the withdrawn animals had a blunted preference for VTA morphine in the presence of DA antagonism, but naïve animals were unaffected by DA antagonism [26,27], indicating a state-dependent component to DA-dependent opioid reinforcement. Demonstration of DA release in the ventral striatum in response to MOR blockage in the VTA [26], indicating that some VTA→ACB DA neurons positively respond to MOR antagonism, further complicates the narrative. Taken together, these data demonstrate a complex interaction between VTA DA neurons and opioid reinforcement.

DA neuron diversity may reconcile the seemingly conflicting findings above, which would imply that opioids have target-specificity, even within DA neuronal nuclei [4,28]. This target-specificity could arise from opioids acting on distinct anatomically-biased midbrain DA neuron subtypes, each with unique projection patterns and functional correspondences [29–33]. One such functional division is a subgroup of DA neurons associated with multiple components of reward, involved in reward prediction error [34–36] and naïve reward response, with a dorsolateral bias [37–39]. In contrast, there is a subgroup associated with aversion-response, which has unique electrophysiologic properties and possibly projection sites, and may be ventromedially biased [40–44]. One interpretation is that the VTA itself has anatomic bias on multiple planes [45], and depending on the exact locations of intervention or recording, opposing results may be seen. Additionally, some studies show that the motor component of opioid action may be dependent on the substantia nigra [46–49], while the rewarding effects depend primarily on the VTA, and some analgesic effects occur at the level of the spinal cord [50–52]. Therefore, work measuring motor changes due to opioid administration may be measuring nigral rather than VTA DA function. One study indicates that some opioid reward function can be attributed to the SNr [14]. Additionally, it has been demonstrated that application of MOR agonists in the caudal VTA, but not the rostral VTA or ACB, results in opioidergic stereotypy behavior [53,54]. That is, only the caudal segment of the VTA resulted in the behavior associated with opioid administration [53], again pointing to subregions within the VTA having somewhat distinct properties. In parallel to this emerging literature on VTA subregions, DA neuron subgroup distinctions between the VTA and substantia nigra [12,28,48, 49,55,56], and even within the VTA and its projections to the ACB [28, 29,32,34] have been shown. The possible relevance of this diversity to help clarify the varied and conflicting studies on the functional properties of DA neurons in opioid reinforcement has yet to be fully explored.

It remains unclear how opioid administration may differentially affect DA subgroups and what are the main projection targets of opioid-recruited DA populations. We therefore devised a screen for DA neuron activation in different opioid administration paradigm to investigate the anatomic specificity of the DA circuitry that is recruited by opioids.

## Results

### Biased distribution of cFos in the VTA following an escalating morphine regimen

**Fig. 1.**
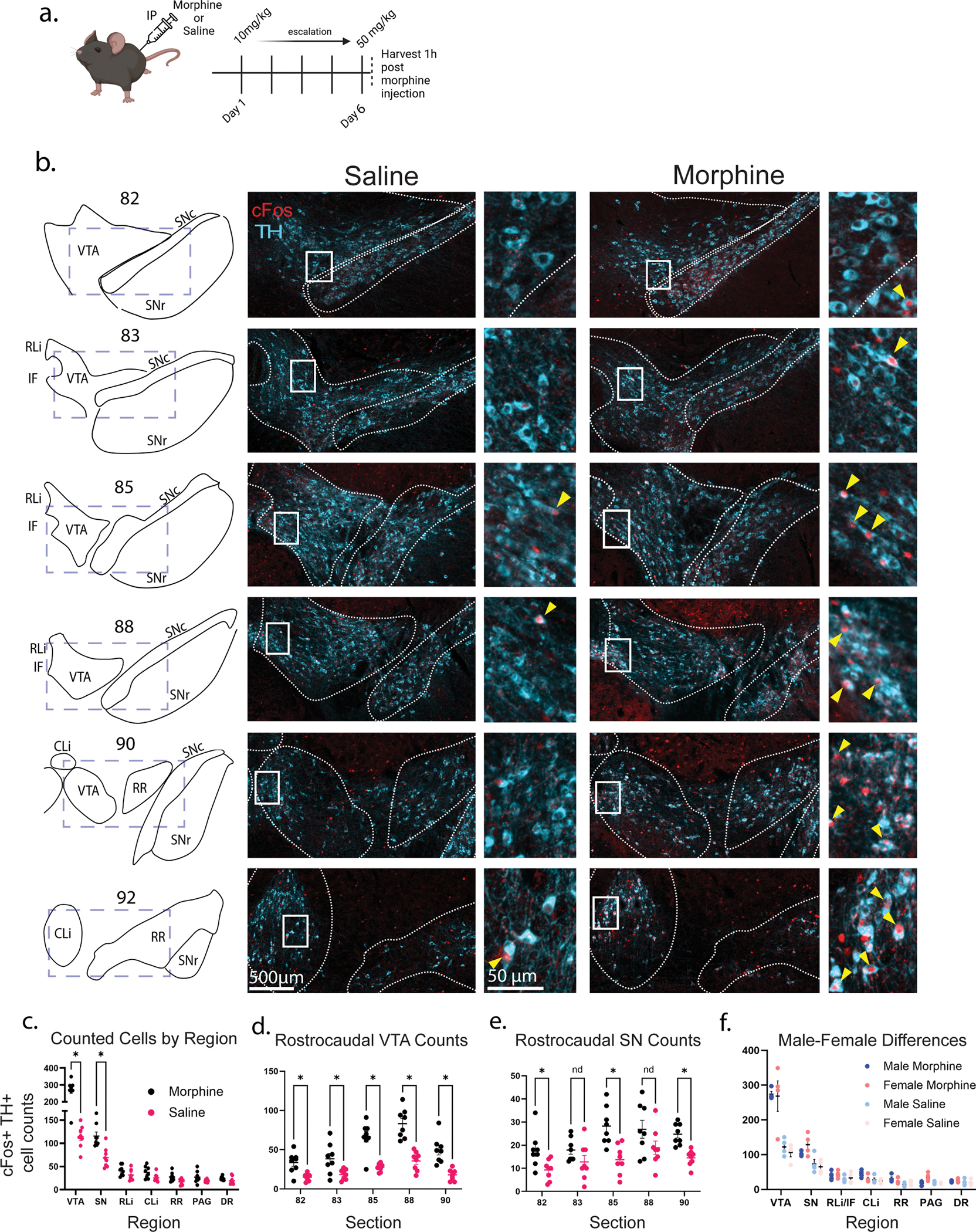
Escalating dose morphine results in anatomically biased cFos activation in DA nuclei. a. Morphine escalation paradigm for C57BL/6 J mice (*n* = 8 morphine, *n* = 8 saline). b. Rostrocaudal dispersal of cFos+ (red) and TH+ (cyan) DA neurons within the VTA and SN in both saline (left) and morphine (right) treated animals. Insets indicate cells which were counted as both cFos and TH staining (yellow arrows) c. Using multiple Mann-Whitney tests, the overall cell counts in the VTA and SN achieved statistical significance (*p* = 0.0022, *p* = 0.0111) while other regions did not. d. Stratified by the section level, VTA had statistically significantly more TH+ cFos+ cells in the morphine condition across all sections (82 *p* = 0.0120, 83 *p* = 0.0281, 85 *p* = 0.0120, 88 *p* = 0.0008, 90 *p* = 0.0008). e. Stratified by section level, SN had statistically significantly more TH+ cFos+ cells in morphine condition in 3 of 5 sections (82 *p* = 0.0199, 85 *p* = 0.0043, 90 *p* = 0.0015). (TH) tyrosine hydroxylase; (tDT) tDTomato; (VTA) ventral tegmental area; (SN) substantia nigra (c) pars compacta, (r) pars reticulata; (RLi) rostrolinear, (IF) interfascicular, (CLi) centrolinear, nuclei raphe; (RR) retrorubral area; (PAG) periaqueductal gray; (DR) dorsal raphe.

To examine the recruitment of DA neurons during opioid administration, we first performed a screen to examine cFos activation within the TH immunopositive midbrain DA neuron population in the presence of high-dose morphine. While a single dose in a naïve animal is a model for the first exposure to opioids, a higher dose after repeated exposure better simulates the development of tolerance and sustained use, which is a more clinically relevant model. We therefore exposed C57BL/6 J wild-type mice to an escalating paradigm of intraperitoneal (IP) morphine, with 10 mg/kg on day 1 up to 50 mg/kg on days 5 and 6, with perfusion 1 h after the final dose of morphine was administered (approximately 45 min after peak morphine) [1,57] (Fig. 1a). We then stained for cFos, a well-studied immediate early gene, as a marker of activity [58], and TH for DA neuron identification (Fig. 1b). Cells containing cFos and TH were counted as activated DA neurons (Fig. 1b).

Broadly, we found that morphine administration recruited more DA neurons in the VTA and SN, but did not significantly recruit TH+ neurons in other DA nuclei such as the rostrolinear nucleus raphe (RL), interfascicular nucleus raphe (IF), central linear nucleus raphe (CL), retrorubral area (RR), PAG, and dorsal nucleus raphe (DR) (Fig. 1c). Aggregated across all sections, using our mixed-effects negative binomial model cFos-activated DA neurons in the VTA of morphine increased 2.359-fold relative to saline treated animals (95 % CI (confidence interval) = 1.907–2.917, *p* = 2.47e-15) (Fig. 1c) and 1.663-fold in the SN (95 % CI = 1.320–2.095, *p* = 1.58e-5) (Fig. 1c). We then examined the VTA (Fig. 1d) and SNc (Fig. 1e) in more detail by counting the cFos^+^/TH^+^ cells across 5 sections throughout the rostrocaudal axis of each. When we further investigated across the rostrocaudal axis using multiple Mann-Whitney tests, the VTA had more cFos+ TH+ cells in the morphine condition across the rostrocaudal axis (*p* ≤ 0.0281) (Fig. 1d), while the SN demonstrated statistical significance only in some sections (82, 85, 90 *p* < 0.05) (Fig. 1e). To evaluate how morphine’s effect may have differed across sections, we evaluated the condition-by-section interaction using an analysis of deviance (see Methods). In both the VTA sections (chisq(4) = 2.8358, *p* = 0.6652) and the SN sections (chisq (4) = 4.666, *p* = 0.3232), the interaction effects were highly compatible with the null hypothesis of a stable morphine effect across the rostrocaudal axis. We therefore conclude that the effect of morphine is relatively consistent across the rostrocaudal axis.

The effect of sex (female-male) on VTA DA neuron recruitment was variable and therefore not statistically significant in any region based on immediate cFos staining (Fig. 1f) according to our mixed-effects negative binomial model. For example, in the saline condition, the female-male effect in the VTA was 0.86 (95 % CI 0.69–1.05, *p* = 0.428), while in the morphine condition, the female-male effect in the VTA was 0.951 (95 % CI 0.720 to 1.256, *p* = 0.725) (Supp. Table 1). We therefore conclude that there are no strong female-male effects in our paradigms.

**Fig. 2.**
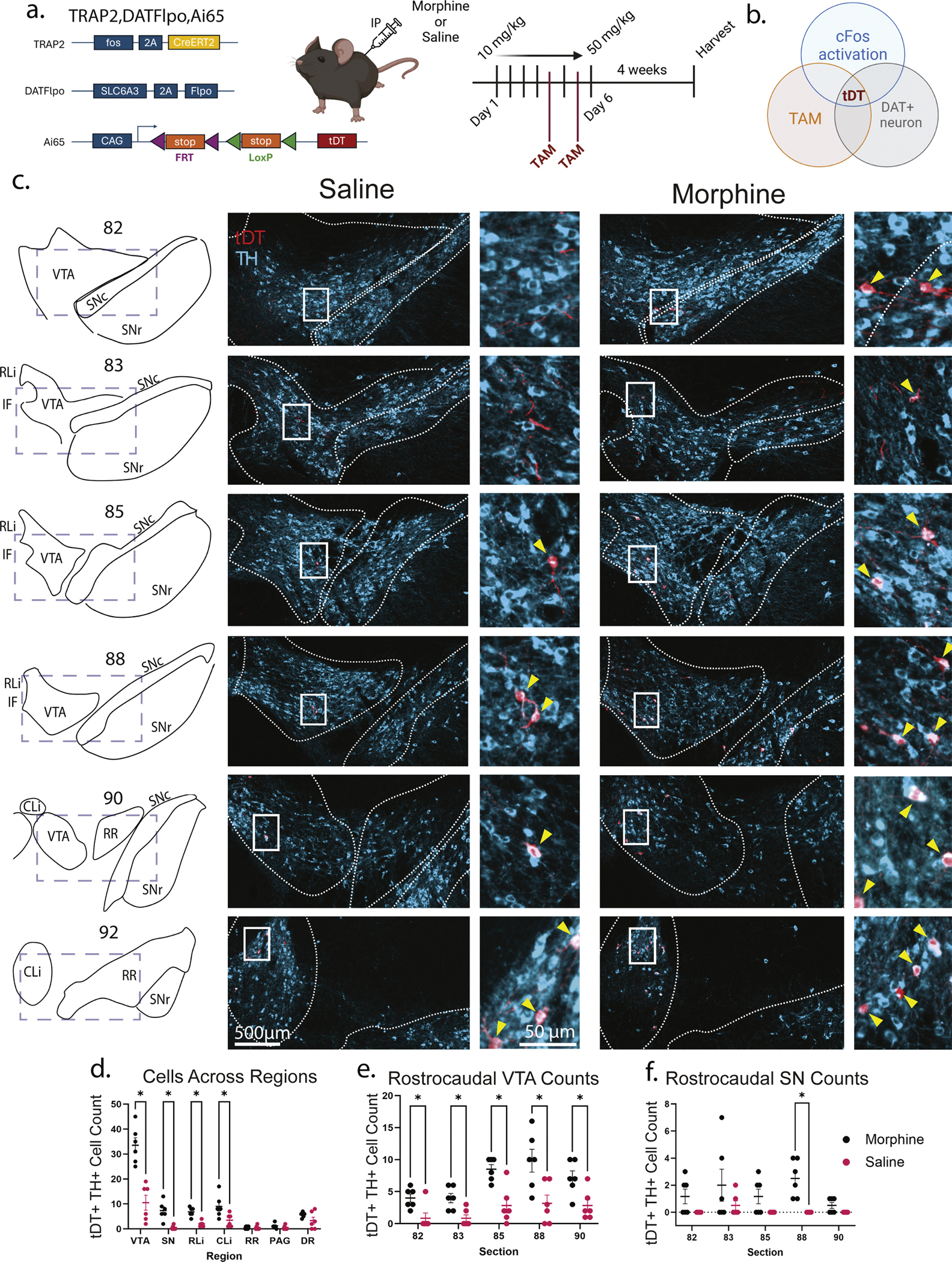
Escalating dose morphine results in anatomically biased capture of cFos-activated midbrain DA neurons. a. TRAP2,DATFlpo,Ai65 mice (*n* = 6 morphine, *n* = 6 saline) received escalating IP injections of morphine across 6 days, with 50 mg/kg delivered on days 5 and 6. Tamoxifen was delivered 6 h prior to morphine on days 5 and 6. b. This allows for capture of solely cFos-activated DAT+ neurons during morphine exposure c. Example captured tDT+ TH+ cells which were counted d. Using mutliple Mann-Whitney tests, statistically significant differences were seen in the cell counts in the VTA (*p* = 0.0022), SN (*p* = 0.0043), RL/IF (*p* = 0.0043), and CL (*p* = 0.0281). e. Across the rostrocaudal VTA, all sections had statistically significantly more tDT+ TH+ cells in morphine treated than saline (82, *p* = 0.0130; 83, *p* = 0.0130; 85, *p* = 0.0108; 88, *p* = 0.0216; 90, *p* = 0.0238) f. SN had statistically significantly more cells in section 88 (*p* = 0.0022).

In order to narrow our scope to DA neurons activated in the presence of morphine, rather than any neuron or even glial cell [59] that may have been activated, we used an intersectional approach to target DA neurons. This approach utilized a combination of the tamoxifen-inducible Cre (CreER^T2^), expressed under control of the *cFos* gene, a DAT-2A-Flpo line, and Ai65D, a Cre and Flp dependent reporter that produces tDTomato (tDT) (hereafter referred to as TRAP2,DATFlpo, Ai65) (Fig. 2a). Therefore, using intraperitoneal injected (IP) tamoxifen, we were able to label DAT-expressing neurons that were active during a particular time window (Fig. 2b). This intersectional genetic technique allowed more flexibility in harvesting the brain, since we were able to visualize captured neurons using a permanent fluorescent marker, rather than by ephemeral cFos expression. Most importantly, tDT filled the axons and was anticipated to provide a more complete picture of the anatomical properties of activated neurons.

Mice were again given the escalating morphine paradigm as above, with tamoxifen administration prior to the 50 mg/kg morphine administration. A single tamoxifen dose yielded insufficient labeling (data not shown), therefore mice were administered tamoxifen on the last two days (Fig. 2a). A single dose of morphine also yielded insufficient recombination (data not shown). We counted tDT+ TH+ neurons across multiple regions, finding statistically significantly more cells captured in not only the VTA and SN, but also RL/CL/IF (*p* ≤ 0.0295 all, multiple Mann-Whitney tests) (Fig. 2d) in the morphine condition than in controls, although the largest cell capture was observed in the VTA. Morphine resulted in a 3.001-fold increase over saline in the VTA (95 % CI = 1.925–4.678, *p* = 1.23e-6), while the SN saw a 5.555-fold increase (95 % CI = 2.673–11.545, *p* = 4.359e-6). The RL/IF had a 2.611-fold increase (95 % CI = 1.516–4.495, *p* = 5.35e-4) and CL had a 2.223-fold increase (95 % CI = 1.304–3.787, *p* = 3.31e-3) in morphine over saline (mixed-effects negative binomial model). Within the VTA, the tDT+ neurons appear notably in the ventromedial paranigral (PN) region, with sparser labeling in the more dorsal VTA regions (Fig. 2c). Investigating the rostrocaudal axis of the VTA, we observed statistically significantly more tDT+ DA neurons in morphine-treated versus saline across all sections (*p* ≤ 0.025) (Fig. 2e); the SN was less consistent, as only one section achieved statistical significance (88, *p* = 0.01; all other sections *p* ≥ 0.552) (Fig. 2f) (Multiple Mann-Whitney tests). When we interrogate the condition-by-section versus constant effect in the VTA, we see no difference due to section level (chisq(4) = 0.5425, *p* = 0.9692), nor in the SN (chisq(4) = 1.6502, *p* = 0.7997). We therefore conclude that the condition effect was relatively consistent across the rostrocaudal axis in both the VTA and SN.

### DA neuron cFos capture during fentanyl administration demonstrates similar anatomic bias

Although morphine is the standard against which opioids are measured, fentanyl is around one hundred times more potent and has a significantly higher affinity to MOR over morphine [60,61]. We accordingly investigated fentanyl-induced DA activation to determine whether morphine and fentanyl impinge on the same DA neuron subgroups, as fentanyl’s increased potency may enhance or alter DA neuron activation.

**Fig. 3.**
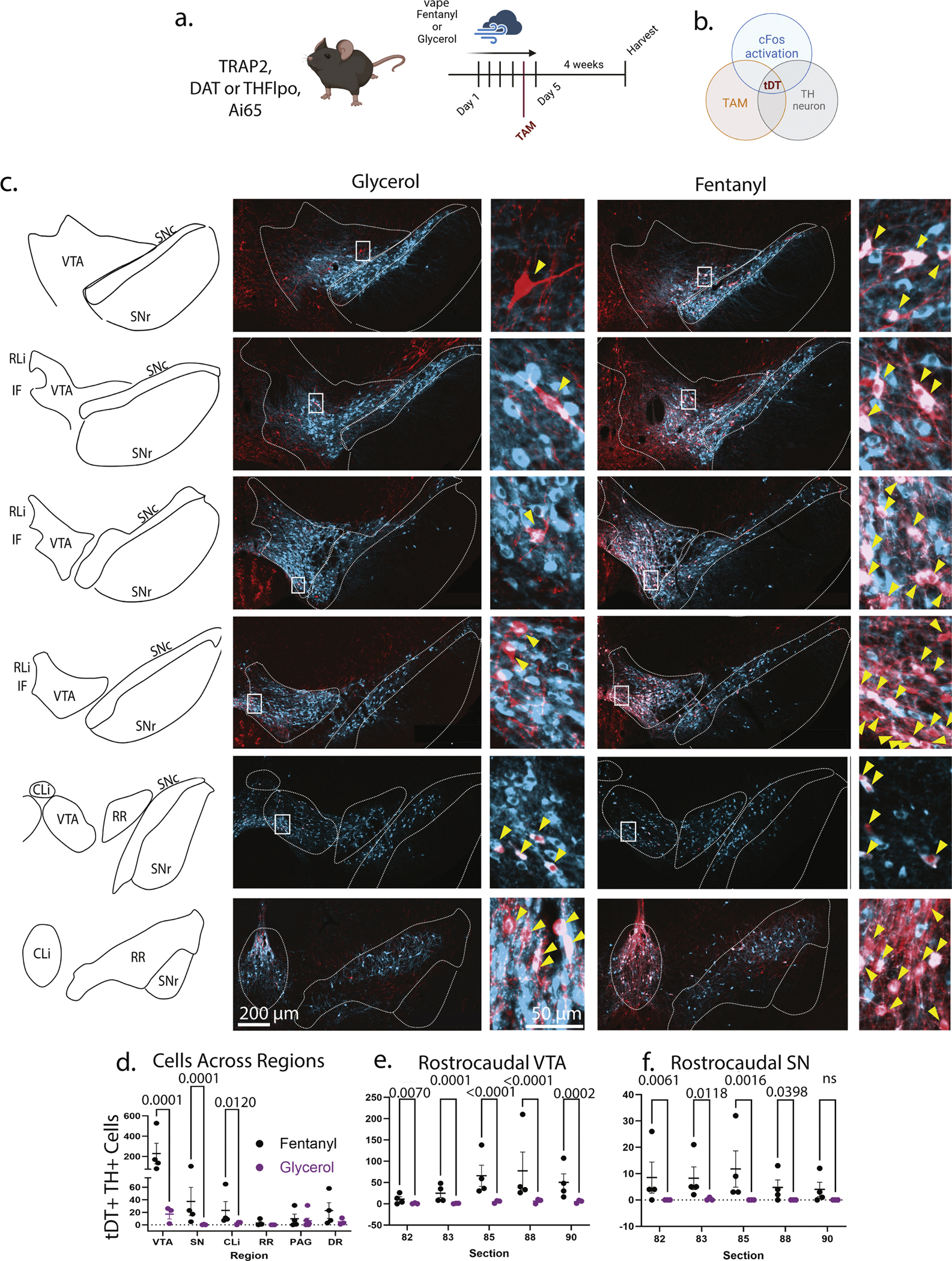
Fentanyl vape administration results in anatomically biased capture of cFos-activated midbrain DA neurons. a. Animals were exposed to a 5 day fentanyl vape self-administration paradigm with an additional 2 h of passive fentanyl on day 5 and tamoxifen delivered 12 h prior to day 5 (*n* = 4 fentanyl, *n* = 3 glycerol) b. TRAP2,DATFlpo,Ai65 or TRAP2,THFlpo,Ai65 mice allow for capture of cFos-active cells expressing DAT or TH during the tamoxifen window c. Example captured tDT+ TH+ cells which were counted d. the effect estimates of fentanyl relative to glycerol in the VTA (fold change 11.94, 95 % CI 3.59–39.71, *p* = 0.0001) SN (fold change 23.81, 95 % CI 5.10–111.11, *p* = 0.0001), and CL (fold change 5.53, 95 % CI 1.46–20.9, *p* = 0.012) demonstrated statistical significance e. in the VTA, section analysis demonstrated that across all levels, the fold change effect was statistically significant (82: fold change 5.99, 95 % CI 1.62–22.10, *p* = 0.007) (83: fold change 13.20, 95 % CI 3.64–47.83, *p* = 0.0001) (85: fold change 10.07, 95 % CI 3.54–28.69, *p* < 0.0001) (88: fold change 9.58, 95 % CI 3.44–26.71, *p* < 0.0001) (90: fold change 7.31, 95 % CI 2.58–20.75, *p* = 0.0002) f. the SN also demonstrated statistically significant effect of fentanyl relative to glycerol across sections 82 (fold change 8.08, 95 % CI 1.81–36.07, *p* = 0.0061), 83 (fold change 5.93, 95 % CI 1.48–23.71, *p* = 0.0118), 85 (fold change 10.91, 95 % CI 2.47–48.21, *p* = 0.0016), and 88 (fold change 4.90, 95 % CI 1.08–22.35, *p* = 0.0398), with only section 90 showing no effect. (mixed-effects negative binomial model).

Fentanyl experiments were performed using a combination of the TRAP2,DATFlpo,Ai65 and TRAP2,THFlpo,Ai65. TH immunopositivity was used to identify appropriate DA neurons for counts, ensuring consistent DA neuron identification across genetic approaches. We found that both transgenic approaches, using THFlpo or DATFlpo, yielded similar results. Mice received 5 days of fentanyl or glycerol (vehicle) self-administration by paired air puff, with a nose poke during a cue light period resulting in a puff of 5 mg/mL fentanyl in the box (Fig. 3a). Associated behavioral data demonstrated self-administration across all groups (Supp. Fig. 9). Tamoxifen capture was done on the final day, which had 1 h of self-administration followed by 2 h of passive administration (Fig. 3a), capturing only TH+ (or DAT+) neurons which were cFos-activated during the fentanyl exposure (Fig 3b). Pure self-administration did not result in sufficient recombination events (data not shown). In the THFlpo strategy, more non-TH-immunopositive cells in non-target regions are visible (Fig. 3c), but these were not included in the analysis, as they represent *Th* mRNA+ cells that have been previously described [29]. Rapid onset and offset of fentanyl function relative to the metabolism of morphine called for a method to better compare longer, continuous fentanyl exposure, rather than a single fentanyl dose. Additionally, allowing animals to self-administer may improve individual titration during the four days of building tolerance, a timeline which was kept stable across morphine and fentanyl. Finally, on the final day during tamoxifen capture, all animals received passive fentanyl exposure to decrease the exposure variability on that day.

Fentanyl largely reproduced the anatomic bias that was observed in the morphine experiments, with soma located mainly in the PN region, with less labeling in the dorsal VTA. RL, RR, PAG, and DR had less cell capture and did not achieve statistical significance (Fig. 3c). The number of VTA, SN, and CL cells activated during fentanyl exposure was much greater than in the control condition, and all three regions had a Mann-Whitney *U* = 0, indicating that all fentanyl counts were higher than all glycerol counts (Fig. 3d). Using a mixed-effects negative binomial model, we found effect estimates throughout the rostrocaudal axis, fentanyl recruited statistically significantly more DA neurons than glycerol in the VTA across all sections (82: *p* = 0.0037, 83: *p* = 0.0002, 85: *p* = 0.0001, 88: *p* = 0.0002, 90: *p* = 0.0009) (Fig. 3e), and in the SN in section 83 only (*p* = 0.0070) (Fig. 3f). However, when we examined the section-by-condition versus constant condition effect across the rostrocaudal axis, we again found no difference in the VTA due to section (chisq(4) = 1.941, *p* = 0.7466) nor the SN (chisq(4) = 1.643, *p* = 0.8011), indicating that the condition effect is stable across sections (mixed-effects negative binomial model). We then wanted to query whether morphine and fentanyl had similar effect. Across the VTA, the effect of morphine (relative to saline) was roughly 0.389 times (95 % CI 0.081–0.697) that of fentanyl (relative to glycerol), while across the SN, the effect of morphine (relative to saline) was roughly 0.517 times (95 % CI 0.035–0.998) that of fentanyl (relative to glycerol) (Supp. Fig. 4) (mixed-effects negative binomial model). These effects align with the established increase in potency and specificity of fentanyl over morphine.

### PAG, DR did not show statistically significant differences in TH+ neuron recruitment across paradigms

The VTA and SN are not alone in recruitment in opioid addiction studies, with some reward processing indicated in other midbrain DA nuclei [40,62,63]. We therefore examined other midbrain nuclei of interest, including the PAG and DR. Though TH+ neurons were captured by all three paradigms (Supp. Fig. 1–3) in these regions, the morphine or fentanyl conditions did not statistically significantly increase this capture in the PAG or DR (*p* ≥ 0.139) (Multiple Mann-Whitney tests). We did not observe an effect of opioids on these regions under our paradigms.

### DA neurons activated by opioids project to specific ACB, amygdalar, and cortical regions

**Fig. 4.**
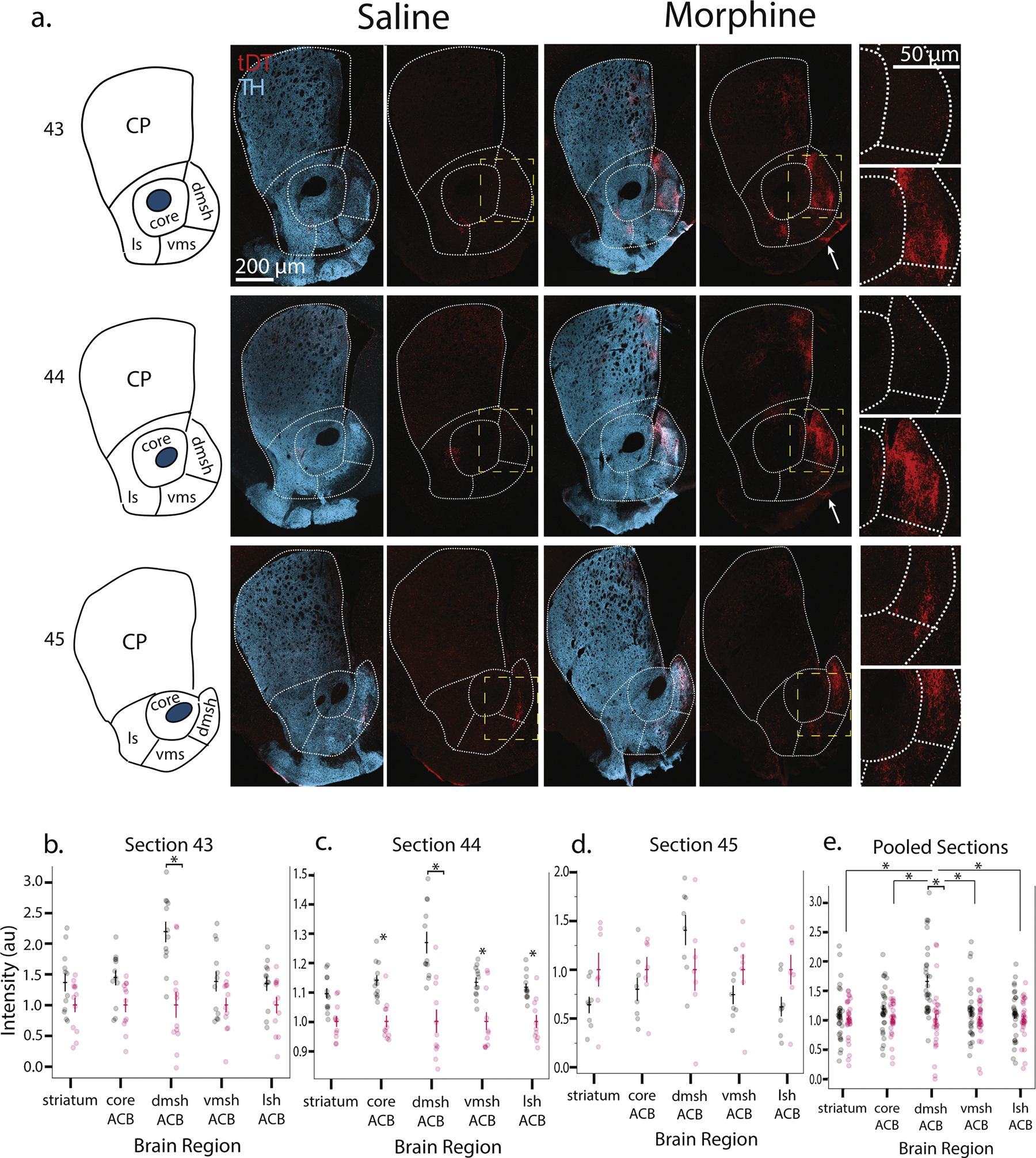
Projection patterns of DAT+ neurons activated during high dose morphine administration a. TRAP2,DATFlpo,Ai65 mice (*n* = 6 morphine, *n* = 6 saline) received escalating IP injections of morphine across 6 days, with 50 mg/kg delivered on days 5 and 6. Tamoxifen was delivered 6 h prior to morphine on days 5 and 6. This allows for capture of solely cFos-activated DAT+ neurons during morphine exposure. Example captured tDT+ projections across the rostrocaudal dorsal striatum, ACB, the BST were quantified bilaterally b. Section 43 had statistically significantly increased intensity in the morphine condition in the dmshACB (*p* = 0.0028) while c. section 44 showed statistically significantly increased intensity in the morphine condition in all regions other than striatum (striatum *p* = 0.0956, cACB *p* = 0.0001, dmshACB *p* = 0.0050, vmshACB *p* = 0.0383, lshACB *p* = 0.0002) (3-way ANOVA). d. section 45 did not have a statistically significant difference in intensity across conditions in any region (*p* ≥ 0.7620, all) e. when pooled across sections, dmshACB showed statistically significant differences in the morphine and saline conditions (*p* = 0.0068). Between regions, the dmshACB was statistically significantly different than all other regions (comparison to: striatum *p* = 0.0024, cACB *p* ≤ 0.0001, vmshACB *p* = 0.0010, lshACB *p* = 0,0008). The striatum was statistically significantly different from the cACB (*p* = 0.0439) and lshACB (*p* = 0.0220) (3-way ANOVA).

Our intersectional approach provides the ability to label activated cohorts of DA neurons in an otherwise unlabeled brain. This allowed us to visualize the projection patterns of the recruited DA neurons. We only examined animals with DAT-2A-Flpo to study the projections of midbrain DA neurons with high cFos activation during morphine administration (Fig. 4). DAT has been shown to be more specific for midbrain DA neuron recombination than TH-driven recombinase drivers [29,64].

We surveyed the whole brain for labeled DA projections and quantified regions of interest, including the prefrontal cortex (PFC), subregions of the ACB, dorsal striatum, bed nuclei of the stria terminalis (BST), and subregions of the amygdala for further analysis. Due to previous work on the ACB in addiction models [15,40,65–71] and the density of projections, we examined a rostrocaudal range of sections (Fig. 4a). The dorsal striatum had much more sparse projections, which likely corresponds to the lesser capture of SN neurons (Fig. 4a), and did not reach statistical significance in any section. The striatal projections tended to be more dorsomedially located (Fig. 4a). Ventrally, ACB showed substantial tDT+ fibers (Fig. 4a). However, ACB subregions were not equally represented (Fig. 4b–e), possibly due to anatomic divisions within the ACB [40]. We saw robust and consistent enrichment of labeled projections in the dorsomedial shell of the nucleus accumbens (dmshACB) in morphine versus saline (43 *p* = 0.0028, 44 *p* = 0.0050, pooled *p* = 0.0068) (Fig. 4b,c,e) (3-way ANOVA). In contrast, increased labeling was only observed in the morphine condition in ventromedial shell (vmshACB), lateral shell (lshACB), and core (cACB) in section 44 (cACB *p* = 0.0001, dmshACB *p* = 0.0050, vmshACB *p* = 0.0383, lshACB *p* = 0.0002) (Fig. 4c) (3-way ANOVA). When we pooled across sections, we saw that only the dmshACB showed statistically significant differences in the morphine and saline conditions (*p* = 0.0068). When we compared regions, the dmshACB was statistically significantly different than all other regions (comparison to: striatum *p* = 0.0024, cACB *p* ≤ 0.0001, vmshACB *p* = 0.0010, lshACB *p* = 0,0008) (3-way ANOVA). The striatum was also statistically significantly different from the cACB (*p* = 0.0439) and lshACB (*p* = 0.0220), in addition to the dmshACB. We saw projections to the BST but these also did not rise above statistical significance (Supp. Fig. 7), likely due to control brains showing some labeling (Fig. 4a). These projection patterns were replicated in the fentanyl condition (Supp. Fig. 5). It appears from these data that the VTA DA neurons projecting to the dmshACB are the most activated by opioid administration, with fewer projections seen to the olfactory tubercle, cACB, vmshACB, lshACB, BST, and dorsal striatum.

We also identified qualitative enrichment in labeled projections to the amygdala, particularly in the basolateral nucleus (BLA) and central amygdala (CEA) but not lateral amygdala (LA) (Supp. Fig. 6). We also saw labeled projections to the PFC (Supp. Fig. 6), which were sparse in the typical VTA to PFC pattern [72]. We saw labeled projections in the medial aspect of the olfactory tubercle (Fig. 4a, arrow). BLA and PFC projection sparsity decreased accuracy of projection density calculations; however, these patterns were observed across all morphine-treated animals.

### DA neurons recruited by opioids are partly comprised of an Aldh1a1+ subtype

**Fig. 5.**
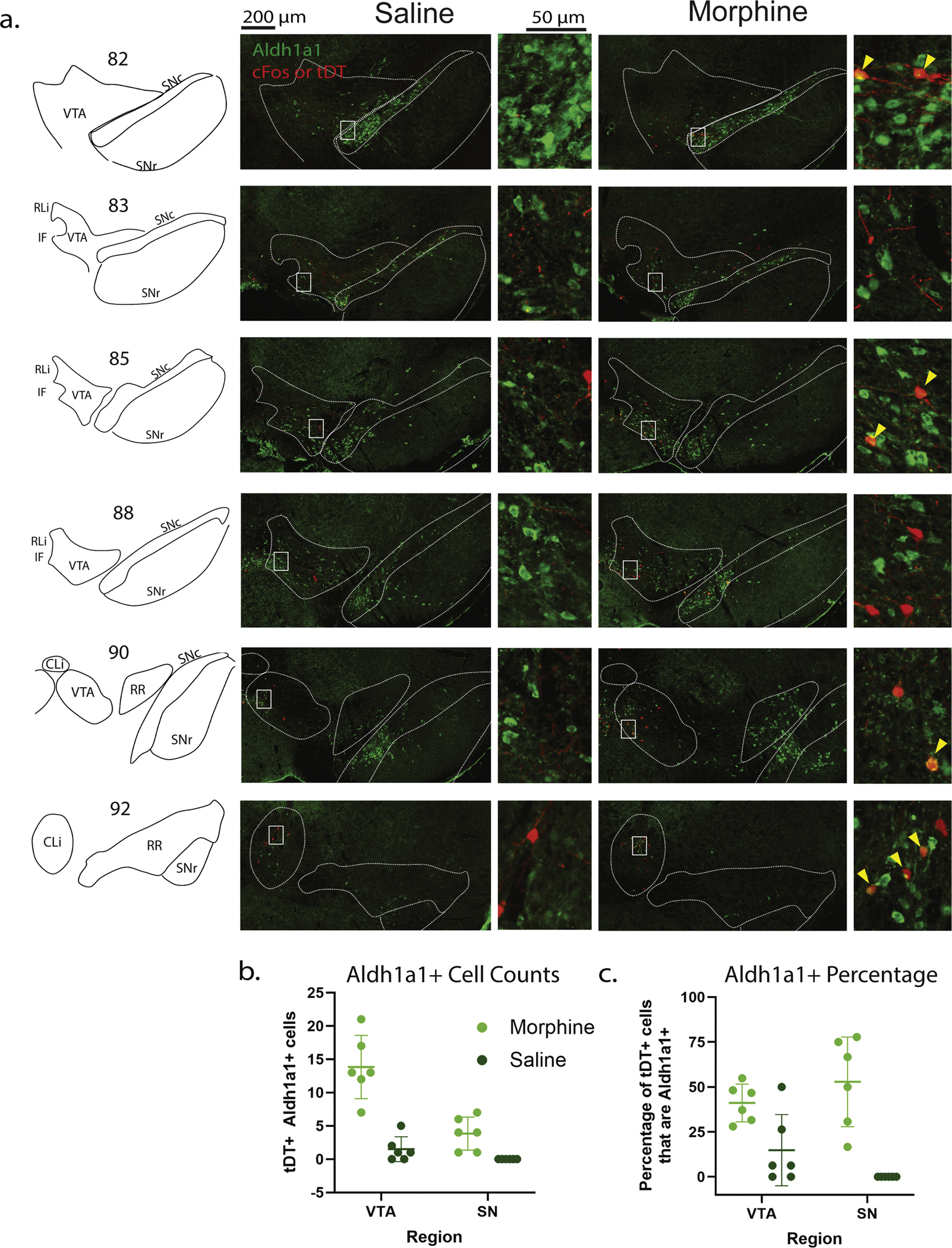
Aldh1a1 marks a portion of captured TH+ cells across conditions. a. Rostrocaudal sections of tDT+ Aldh1a1+ cells (yellow arrows) in VTA and SN b. showing the counts of DA neurons that costained Ald1a1+ and tDT+ cells per animal (mean, standard deviation) c. Percentage of tDT+ neurons co-labeling with Aldh1a1+ per animal (mean, standard deviation).

Of the TH+ cells in the VTA seen in both immediate (Fig. 1c) and transgenic capture experiments (Fig. 2c), there was a clear bias towards the PN segment of the VTA. Due to this anatomic bias, we tested the hypothesis that the activated neurons would be enriched in Aldh1a1 expression, as previous studies have shown a similar PN bias in Aldh1a1+ VTA DA neurons [28,29,73,74]. Additionally, the dmshACB-focused projection mirrors the documented Aldh1a1+ VTA DA projection pattern [29]. We therefore stained for Aldh1a1 and examined all sections for co-labeling with our activated population (Fig. 5a). First examining the immediate cFos staining (Supp. Fig. 8a), we saw that an average of 90.13 (±21.77) VTA DA neurons costained Aldh1a1+ out of an average of 271.125 (±58.31) cFos+ VTA DA neurons in the morphine condition. In contrast, an average of 33.88 (±9.64) VTA DA neurons costaining Aldh1a1+ out of an average of 114.38 (±24.44) cFos+ VTA DA neurons was observed in the saline condition (Supp. Fig. 8b). In the SN, a mean of 62.00 (±21.43) DA neurons costained Aldh1a1+ out of an average of 115.63 (±25.22) cFos+ DA neurons were seen in the morphine condition. In the SN saline condition an average of 31.50 (±9.53) DA neurons costained Aldh1a1+ out of an average of 69.13 (±21.60) cFos+ neurons were observed (Supp. Fig. 8b). We therefore found that, in the immediate cFos staining experiment in the VTA (Supp. Fig. 8a), an average of 33.31 % of cFos+ DA neurons were also Aldh1a1+ in the morphine condition, while in the saline condition, an average of 29.21 % costained (Supp. Fig. 8b). Using these proportions we calculated that there were approximately 20 % greater odds of a cFos+ cell being Aldh1a1+ in the morphine over saline condition in the VTA in the immediate staining experiment (95 % CI of condition effect 1.02–1.44, quasibinomial generalized linear model). In the SN in the immediate cFos staining experiment, an average of 52.68 % of cFos+ TH+ neurons in the morphine condition were also Aldh1a1+, while in the saline condition, an average of 45.8 % were Aldh1a1+ (Supp. Fig. 8b). There are approximately 30 % greater odds of a cFos+ cell being Aldh1a1+ in the morphine over saline condition in the SN in the immediate staining experiment (95 % CI of condition effect 1.04–1.67, quasibinomial generalized linear model). Together, in the immediate staining condition we saw a modest enrichment in Aldh1a1+ DA neuron capture.

Using our transgenic capture method (Fig. 5a), we found that in the VTA, in the morphine condition, we saw that a mean of 13.83±4.75 were Aldh1a1+ out of an average 33.50±7.42 tDT+ VTA DA neurons. In contrast, in the saline condition we counted 1.50±1.87 Aldh1a1+ out of an average of 10.50±7.31 tDT+ DA VTA neurons (Fig. 5b). Using our transgenic capture method, we found that in the VTA, an average of 41.06 % of tDT+ cells were also Aldh1a1+in the morphine condition, and in the saline condition only an average of 14.83 % were Aldh1a1+ (Fig. 5c). Meanwhile the SN reproduced at 52.3 % in the morphine condition, with 0.00 % of SN captured cells being Aldh1a1+ in the saline condition (Fig. 5c). In the transgenic experiment, there are approximately 180 % greater odds of a tDT+ cell being Aldh1a1+ in the morphine over saline condition in the VTA, although with less certainty (95 % CI of condition effect 0.87–9.33, quasibinomial generalized linear model). In the SN, we counted a mean of 3.83±2.48 Aldh1a1 costaining DA neurons out of an average of 7.33±3.67 tDT+ in the morphine condition, while in the saline condition we counted a mean of 0.00±0.00 Aldh1a1+ SN DA neurons out of an average of 0.50±0.83 tDT+ cells (Fig. 5b), with insufficient counts to provide odds estimates. In both immediate and transgenic capture, the vast majority of identified Aldh1a1-costaining DA neurons were in the VTA over the SN, and in the morphine condition over the saline condition. Taken together, we describe that approximately one-third-of the opioid-activated DA neurons of the VTA, and about half of the opioid-activated SN neurons, are Aldh1a1-expressing, although in all cases the tDT+ (or cFos+), Aldh1a1+ cell numbers are substantially greater in the VTA. While Aldh1a1 labels a substantial portion of opioid-activated DA neurons, it does not entirely encapsulate the recruited population.

As an effort to compare to another documented VTA DA subgroup marker, we then stained separate sections from the same brains for Calb1, which has been shown to identify several subtypes of VTA DA neurons [28]. There was substantial co-staining, as expected, as this marker is expressed in a large fraction of VTA neurons and some dorsal SN neurons (Supp. Fig.10) [55].

## Discussion

Continued developments in the field of DA neuron heterogeneity suggest that isolation of specific DA neuron subsets engaged in complex behaviors, such as substance use, is increasingly important to our understanding of the precise circuits involved. We therefore began by asking if a demonstrable anatomic bias in midbrain DA neuron recruitment is observed during high-dose morphine administration. We determined that cFos-activated TH+ cells did not uniformly distribute across the VTA, SN, and other DA nuclei, but rather, were more narrowly focused in the ventromedial paranigral region of the VTA. This distribution was replicated using an intersectional cFos-TRAP transgenic method, which allows for whole-cell labeling, although at a lower efficiency. The somatic pattern of the transgenic capture recapitulated the immediate cFos staining, a strong indication that, though this method captures fewer cells, they remain representative. Additionally, ventromedial VTA cell capture was also seen in a model involving fentanyl, which more selectively binds MORs, indicating that it is the effects of MOR agonism that are captured by this method. To assign a molecular identity to the activated group of DA neurons, we co-stained for Aldh1a1, a marker for a documented subgroup that has soma biased towards the PN VTA and projects to the mshACB [28,29,55]. We determined that, within the VTA, this subtype accounts for 33.3–41.3 % of captured DA neurons. It therefore remains an important open question what other DA neuron subtypes are expressly activated by opioid administration [55]. Our method allowed for examination of not only the soma location of the DA neurons activated by opioids, but also their projection patterns. Within the ACB, this opioid responsive population projected mainly to the medial shell (mshACB), compared to the cACB or lshACB, and within the mshACB, was more predominantly dorsal. Additionally, this population projects to the BLA and to the PFC. The specificity of these projections, especially within the dmshACB, indicates that the subset of DA neurons maximally recruited by opioids is more precise than previously thought.

Our projections to the mshACB subregion recapitulate several opioid reward studies, likely indicating capture of the VTA DA population responsible for the reward behaviors associated with opioid administration [10,75]. In the VTA, heroin has been shown to recruit medial DA neurons, which project and release DA in the mshACB [10]. Chronic self-administration of heroin increased extracellular DA in the mshACB over the core of the ACB [76], as did morphine administration [77]. Moreover, antagonizing DA in the shell, but not the cACB, impaired conditioned place preference (CPP) to morphine [78]. This is all in contrast, however, to robust evidence of plasticity changes in the cACB during opioid escalation paradigms. These include increasing spine and dendritic densities in local medium spiny neurons (MSNs) in response to morphine [79], and a reciprocal loss of dendritic arborization in withdrawn animals [80], demonstrating local cACB neural plasticity to opioid use and withdrawal. Beyond this, a long-term delayed increase in glutamatergic receptor density in cACB MSNs following escalating morphine [81], and alterations in DA uptake due to opioid exposure [82] further indicate some cACB regulation during opioid administration and withdrawal. While we cannot rule out the cACB as an important contributor to the reward learning involved, in our paradigm, we find that the dmshACB is robustly innervated by captured DA neurons, with fewer projections observed to the cACB or lshACB. This aligns with the mshACB coding for reward from addictive substances [68,83].

Our data pose a conundrum; while our projections are observed most robustly in the dmshACB, a region studied for reward, our somatic bias to the PN VTA is more consistent with aversive-responsive DA neurons [44]. One plausible explanation for the discrepancy is the cellular complexity within the PN VTA, which may house both aversive-responsive as well as reward-responsive DA neurons. The heterogenous DA neurons of the VTA are heavily intermingled [55,56], giving rise to the possibility that our captured subgroup, substantially Aldh1a1+ and present in the PN, is not well represented in recording literature [42,44,84]. Due to the high specificity of our projection, which aligns with reward-responsive axons in the dmshACB [85,86] it seems most likely that there is a rewarding, albeit PN-biased, VTA DA neuron subgroup. This is consistent with a finding that activating dmshACB, but not vmshACB, neurons resulted in place preference [87]. Our data therefore align with the idea that the dmshACB is a reward hotspot, including in the case of opioids, while the vmshACB and lshACB may have more complex functions [40,42,85],.

The identification of Aldh1a1 as a putative marker of a subpopulation, or portion thereof, of VTA DA neurons derepressed by opioids reinforces the continued importance of investigating DA neuron subtype functionality. Although in the SN, Aldh1a1 has been proposed as a marker for vulnerable neurons in Parkison’s models [88], less is known about the function of Aldh1a1+ population in the VTA. Generally, within a DA neuron, the function of Aldh1a1 is hypothesized to be protective against the buildup of toxic aldehydes, and its absence results in dysregulation of DA neuron signaling [89,90]. Within the circuit, it appears that Aldh1a1+ DA neurons projecting to the striatum impinge on MOR-dense areas, and Aldh1a1 knockout resulted in decreased MOR in striatal patches [90]. Although these findings were in the dorsal striatum and SN DA neurons, the pattern was observed throughout the ventral striatum and the knockout would, by necessity, include VTA DA neurons, although this group was not intensively investigated. Within the ACB, the Aldh1a1+ VTA DA neurons project primarily to the dmshACB, which also appears to be a reward hotspot [29,84,86]. One pathway that Aldh1a1 is involved in intracellularly is retinoic acid synthesis, following which retinoic acid may be utilized as a signaling molecule to downstream neurons [90,91]. It is this retinoic acid signaling thought to modulate the MOR expression on downstream MSNs in the striatum [90]. However, more work must be done to determine the function(s) of Aldh1a1 in this subpopulation in the VTA, as well as the impact of Aldh1a1+ neurons in this circuit.

The canonical circuit for opioid activation of VTA DA neurons is via disinhibition, or relief from tonic inhibition by the GABAergic neurons of the RMTg [22,92–94]. The VTA receives substantial GABAergic input both locally and distally, but local-origin and RMTg GABA neurons are more likely to synapse on VTA DA neurons [95,96]. Further, RMTg-evoked inhibitory post-synaptic currents (IPSCs) were over twice as sensitive to morphine modulation as IPSCs from the ACB or more rostral VTA [97], and RMTg GABA neurons were hyperpolarized by MOR agonism [98], making the RMTg an important candidate for VTA DA regulation. Lateral (and dorsal) VTA targeting of RMTg Vgat+ projections results in conditioned place aversion [92,97,98], in line with other findings of RMTg activation opposing VTA DA activation [99]. Lateral VTA DA neurons project to ACB core or lshACB [29,100,101]. Due to the above functional studies, the field has primarily focused on the lateral VTA, or parabrachial (PBP), as a key target for RMTg axons. Therefore, our findings of enriched projections in the dmshACB at least partly contradict the previous model. Supporting our results is previous work showing RMTg GABAergic inputs to the VTA target both PBP and PN regions, but are biased towards the PN [96]. It is therefore possible that RMTg disinhibition by opioids more robustly affects PN VTA, resulting in the pattern of labeling observed.

Another source of opioid reward may come from projection to PFC, specifically in the infralimbic area (ILA). Optogenetic stimulation of VTA DA terminals in the ILA induced reinforcement behavior [102], suggesting that this projection carries rewarding properties. Conversely, ILA neurons inhibited medial VTA neurons [103], while projections from the ILA to the VTA can bidirectionally modulate DA neural activity [104], demonstrating feedback. Further, cortical neurons of the ILA project to the mshACB and are recruited in reward states [105]. It is therefore of great interest that the VTA to ILA projection was captured in our screen and may indicate that this projection is an important modulator for opioid-recruited circuitry. However, since DA axons in the PFC have been shown to respond to both rewarding and aversive signals, functional work will have to be done to confirm the valence of this projection [42,106–108].

A further interesting finding was the projection to the BLA, which has also been implicated in aversive signaling [42,109,110] and anxiety-like behaviors [111]. One explanation for the recruitment of the BLA is an aversive component to opioids. Indeed, one study found that, despite continuing to self-administer fentanyl, rats consistently made distress vocalizations while under its influence [112]. Our data appear to capture solely opioid action, not withdrawal, yet the possibility remains that even internal to opioid use, there is an aversive component not captured by seeking behavior. An alternative is that this circuitry is multivalenced and is a component of reward. The BLA, in particular, has also been associated with reward calculations [113], especially comparisons between rewards [114], and fear extinction precipitated by DA [115]. Further, BLA DA axon activity, as well as the expression of both reward and aversive behavior are, in some cases, state-dependent [109]. Yet another possibility is that there are separable reward and aversive VTA DA projections to the BLA, but functional studies to confirm the valence of the VTA to BLA projection will be needed.

Soma in the PAG and DR were captured, but with uncertainty, in contrast to a previous study showing DR DA neuron response to opioids [62]. This could indicate that perhaps the particular paradigm of incentivization of the opioid administration used in a previous study [62] may be critical to recruit these DA neurons. Alternatively, DR DA neurons have also been shown to have elevated cFos during increased wakefulness, and given that all animals in this study were roused for injections, this could result in cFos capture of a DA population across both morphine and saline conditions [116,117]. In addition, DR DA neurons were also found to be activated by salient stimuli regardless of valence [117], which may indicate that even saline injection is salient at the level of the DR and resulted in some cFos capture, thereby obscuring the potential opioid recruitment.

An important consideration for this study was to confirm that cFos expression readily mimics activity in DA neurons across midbrain nuclei. This is quite evident from the literature, which shows that DA neurons in both the SN and VTA are capable of robustly expressing cFos [118,119]. A second concern was whether disinhibition of DA neurons would be sufficient to induce cFos expression, and indeed, this has been demonstrated by optogenetically activating long-range GABAergic inputs to local VTA GABA neurons, following which cFos expression was observed in VTA DA neurons [96]. Other midbrain DA regions, such as DR DA neurons, also robustly induced cFos during testing of CPP in response to reward paradigms [62], showing that cFos is a reliable marker for activated midbrain DA neurons. Although the efficiency of the transgenic method is lower (around 10 %, 95 % CI 3.3 %–15.8 %, with 9.3 % in the VTA), the similar somatic distribution within the DA nuclei across immediate and transgenic methods indicates that the sparser recombination events remain representative. In the case of fentanyl, further work using intravenous self-administration paradigms may allow for better regulation of blood levels of fentanyl to corroborate and extend these findings. Additionally, newer techniques which allow for labeling of inhibited neurons in a similar manner to cFos may be a window into opioid-related states of DA neurons [120]. Notwithstanding these limitations, our findings clarify the specificity of DA neuron activation in the presence of systemic opioids and present target subtypes and subregions for further study.

The relatively long time window afforded by tamoxifen could theoretically lead to the capture of DA neurons responding to morphine withdrawal at the end of exposure. However, this does not appear to be the case, as the pattern of somatic capture in our immediate cFos condition (Fig. 1), in which animals were euthanized while still intoxicated with morphine, mirrors our capture in the transgenic replicate (Fig. 2). Additionally, the projection pattern to the dmshACB is highly specific and widely considered a hotspot for rewarding DA signals. Nonetheless, our findings should be corroborated by functional methods to confirm the valence of the labeled circuits.

Despite the long-held connection between VTA DA neurons and opioid addiction, little has been done to address the growing literature on DA neuron heterogeneity in this context. As a contribution to disentangling the issues of specific circuit recruitment and DA-subgroup susceptibility to opioid activity, we examined both the soma location and projections of these cells. Prospective labeling of the projections of morphine-recruited DA neurons has not previously been described, and here our work adds specificity to the question of circuit recruitment by opioid exposure. The high specificity of DA neuron recruitment emphasizes that targeting DA neurons for therapeutic approaches must take into consideration the most opioid susceptible population(s).

## Materials and methods

### Mice

C57BL/6 J adult mice from Charles River were used as a background strain to triple transgenic Fostm2.1(iCre/ERT2)Luo/J, DAT2AFlpo - B6, 129S-Gt(ROSA)26Sortm65.1(CAG-tdTomato)Hze/J, hereafter referred to as TRAP2,DATFlpo,Ai65. In some experiments where noted TRAP2, TH2AFlpo,Ai65 animals were used. Males and females were represented at 1:1 in all groups.

**Table:** 

Strain	Source
030,323 (TRAP2)Fostm2.1(icre/ERT2)Luo/J	Jackson Laboratories
TH-2A-Flpo	Awatramani Laboratory / Northwestern University Transgenic and Targeted Mutagenesis Laboratory, [29]
021,875 (Ai65) B6;129S-Gt(ROSA) 26Sortm65.1(CAG-tdTomato) Hze/J	Jackson Laboratories
DAT-2A-Flpo	Awatramani Laboratory / Northwestern University Transgenic and Targeted Mutagenesis Laboratory, *publication forthcoming*

### Morphine administration paradigms

Morphine administration was given IP at increasing concentrations from 10 mg/kg to 50 mg/kg for 6 days, that is: day 1: 10 mg/kg, day 2: 20 mg/kg, day 3: 30 mg/kg, day 4: 40 mg/kg, day 5: 50 mg/kg, day 6: 50 mg/kg, with tamoxifen T5648–1 G from Sigma diluted in pure corn oil and delivered by intraperitoneal injection at 150 mg/kg 6 h prior to the final two (day 5, day 6, both 50 mg/kg) morphine injections. In pilot experiments, some animals received only one tamoxifen injection 6 h prior to day 6 (50 mg/kg morphine), but these had insufficient numbers of labeled cells. Animals for cFos antibody staining were timed perfused 1 h after their final morphine dose (Shown in Fig. 1) while TRAP2, DATFlpo,Ai65 animals were perfused at least 4 weeks after their final morphine dose (Shown in Fig. 2). To ensure sufficient arbor filling by the tDT expression, mice recovered for 4 weeks prior to harvest.

### Fentanyl administration paradigm

The chambers for vapor self-administration were built completely in-house. The apparatus is composed of a hermetically sealed transparent acrylic box (14 cm × 20 cm × 23 cm) with two ports: an inlet and an outlet. A flexible tube connects the inlet of the box to a vaping vaporizer; another tube connects the outlet to a HEPAA filter and then a vacuum line. The tank of the vaporizer is filled with either drug or vehicle. A negative pressure system sucks the vapor into the chamber when the vaporizer is active. The vapor flow is regulated so one vapor puff is cleared within 60 s. Two nose poke devices are attached to the chamber. One nose-poke device is active and poking it may result in activating the vaporizer and its delivery into the chamber, the other nose poke device is inactive. A Med Associate system handled the control and data acquisition of the apparatus. Each apparatus is placed in a ventilated, sound attenuating and dark cabinet. Fentanyl administration started with 5 days of 1 h fixed-ratio 1:1 (one correctly paired nosepoke to one puff of fentanyl), with tamoxifen delivered 12 h prior to a combined 1 h fixed ratio (FR1) and 2 h of passive fentanyl, which puffed every 2–7 min. The fentanyl solution concentration was 5 mg/ml. Each puff was 2 s long, with 1 puff per dose. The airflow rate in the chamber was 2 liters/min and the vaporizing duration was 2 s. 5 mg/ml fentanyl HCl was dissolved in a vehicle consisting of 20 % propylene glycol and 80 % figol. Fentanyl was obtained from National Institute on Drug Abuse (NIDA), Intramural Research Program Pharmacy, Baltimore, MD, USA. The duration of drug vapor in the chamber depends on airflow rate, power setting of the vaporizer, and vaporizing time. In our experiments, these were adjusted to allow drug clearance within 1 min after each vapor delivery of 1 puff. These tanks are courtesy of the Center for Translational Pain Development. Pure self-administration paradigms did not yield capture of active neurons significantly above controls, nor did single morphine doses (data not shown).

### Immunohistochemistry

Stainings were completed in accordance with Luppi et al. [121]. Adult brains were perfused and fixed overnight with 4 % PFA-PBS, cryoprotected in 30 % sucrose-PBS solution, frozen in dry-ice and sectioned at 25 μm on a microtome. Sections were rinsed in PBS and blocked in 5 % normal donkey serum in PBS 0.3 % Triton X for 30 min at room temperature (overnight at 4 °C for staining with goat anti-Aldh1a1), then incubated with 1:1000 primary antibodies (see chart below) diluted in blocking solution overnight at 4 °C. The following day, sections were rinsed in PBS and incubated with secondary antibodies at 1:250 and DAPI for 2 h at room temperature, followed by PBS rinse and mounting. Slides were ultimately dried and coverslipped with Gelvatol.

**Table of Antibodies Used:** 

Antibody	Source	Cat. No.
Goat Polyclonal anti-Aldh1a1	R&D Systems	Cat# AF5869; RRID: AB_2,044,597
Rabbit Polyclonal anti-RFP	Rockland	Cat# 600–401–379; RRID: AB_2,209,751
Chicken anti-TH	Abcam	Cat# ab76442
Mouse monoclonal anti-CALBINDIN-d-28K	Sigma	Cat# C9848; RRID: AB_476,894

### Cell counting and projection quantification

All processes were done in accordance with Luppi et al. [121]. Fluorescence images were acquired on a Leica DM5000 and Olympus Slide Scanner VS120 at 10x and 20x resolution. Cropped images captured the width of the brain and height of all regions of interest, ranging 30,000–50,000 pixels. We referred to the mouse brain Allen Reference Atlas (by Wiley) and Paxinos G. & Franklin K. B (second edition by Academic Press) to identify brain section levels and establish anatomical boundaries in conjunction with TH expression. Multi-channel images were processed in CellSens (Olympus) and Fiji (NIH) software to draw boundaries and perform cell counts. All counted cells were DAPI+ and all DA neurons counted were TH immunopositive. In cases where THFlpo animals were used, any cell not TH immunopositive was excluded. Cell counts were performed on 2–8 brains per group and 7 sections per brain. Counts were previously verified by 2 raters, which had 95 % agreement (data not shown). Statistics were performed in GraphPad PRISM or R.

### Glossary

**Table:** 

Region label	Definition as used for quantifications
ACB	Nucleus accumbens, Allen Atlas • Core, Paxinos Atlas • Medial shell (ms), dorsomedial shell (dms), ventromedial shell (vms), lateral shell (ls), previous publications
BLA	Basolateral amygdalar nucleus, Allen Atlas • BLAa anterior part • BLAp posterior part • LA lateral amygdalar nucleus
BST	Bed nuclei of the stria terminalis
CEA	Central amygdalar nucleus, Allen Atlas
CI	Confidence interval
CL	Central linear nucleus raphe caudal part, sections 90–92, Allen Atlas
DA	Dopamine
DR	Dorsal nucleus raphe, Allen Atlas
MOR	M-opioid receptor
MSN	Medium spiny neuron
PAG	Periaqueductal gray, Allen Atlas
PBP	Parabrachial area, Allen Atlas
PFC	Prefrontal cortex, previous publications, refers to CTX cerebral cortex, Allen Atlas • PL prelimbic area • ILA infralimbic area • Layers, Allen Atlas
PN	Paranigral area, Allen Atlas
RL	Rostral linear nucleus raphe, central linear nucleus raphe, and interfascicular nucleus raphe in sections 83–88, Allen Atlas
RR	Midbrain reticular nucleus, retrorubral area, Allen Atlas
SN	Substantia nigra, including pars compacta, lateralis, and reticulata, Allen Atlas
VTA	Ventral tegmental area, Allen Atlas

### Section to bregma coordinate conversion

**Table:** 

Section Number	Bregma Value
36	1.845 mm
43	1.145 mm
44	1.045 mm
45	0.945 mm
49	0.545 mm
75	−2.055 mm
82	−2.88 mm
83	−2.98 mm
85	−3.18 mm
88	−3.455 mm
90	−3.68 mm
92	−3.88 mm
98	−4.455 mm

### Analysis

For the cell counts, we used GraphPad PRISM to perform Mann-Whitney U nonparametric rank-sum testing with Holm-Šídák multiple comparison corrections to determine significance with *a* = 0.05 when data was sufficiently powered to use rank-sum testing. In this and exception cases, we also fit a mixed-effects negative binomial model using restricted maximum likelihood to quantify the effects of morphine (or fentanyl) exposure on tDT+ cell counts in each region. We included region, condition (saline vs. morphine), and their interaction as fixed effects, and we included a random intercept for each mouse. We fit a negative binomial model using maximum likelihood for models that did not model multiple regions at once, and we excluded region as a variate. Since the negative binomial model is a multiplicative model that relies on a log-link function, groups with zero cell counts (e.g., the SN saline condition) could not be modeled; thus, to facilitate model convergence, we modeled count+1 rather than the raw counts. In a couple of instances, the dispersion model was not necessary, which caused convergence issues; in these cases, a Poisson likelihood was used. *P*-values and CIs were calculated based on normal assumptions. For presentation purposes, the condition estimates and CIs for each region were exponentiated so that they could be (approximately) interpreted as fold changes. This method was also applied to male/female comparisons. Finally, we evaluated the effect of the condition-by-section interaction using analyses of deviance with Chi-squared assumptions; for these comparisons, the generalized mixed-effects models were fit using maximum likelihood rather than restricted maximum likelihood. Due to concern for insufficient power to reject the null via multiple Mann-Whitney in the fentanyl condition, only the mixed-effects model was used. For Aldh1a1 comparisons, we estimated the effect of condition (opioid vs. control) on the proportion of Aldh1a1+ cells that expressed cFos using a quasibinomial generalized linear model. This model appropriately models proportions, including when 0′s and 1′s are present in the data, and estimates the effect on the log-odds scale; we exponentiated these effects to obtain odds ratios and 95 % CIs via profile likelihoods. For projection intensity values, we ran a 3-way ANOVA (section X region X condition) where section and region are a within-subjects variable and condition is independent. Post-hoc tests compared condition effects across regions and sections.

The projection comparisons were quantified using the ASAP protocol developed in our lab (James Lyman, Natalia Lopez Gonzalez del Rey https://www.protocols.io/view/pipeline-for-image-processing-and-quantification-o-4r3l2qojpl1y/v1). Briefly, single-channel TIFF files are warped to the corresponding Allen Reference Atlas section (bilateral, for *n* = 12 samples per group per section) and regions of interest (ROIs) are then imposed. Sections with poor matching to the Atlas section were removed, blinded to condition. The pixel density, a proxy for axon density, is determined in Fiji and calculated per ROI, accounting for ROI size. Each image also had two background ROIs measured, and each ROI of interest was normalized as follows: (ROI intensity – background intensity)/background intensity. A 3-way ANOVA (section X region X condition) where section and region are a within-subjects variable and condition is independent was run on these results. The intensities were then normalized for cross comparison by setting the mean of each saline group =1 and all other values were adjusted by 1, correspondingly. CEA was excluded due to the presence of a few confounding labeled soma.

### Software used

**Table:** 

Software	Source
CellSens	Olympus
GraphPad Prism	Insightful Partners
Fiji (ImageJ)	NIH
R	R Foundation for Statistical Computing

## Supplementary Material

1

2

Supplementary materials

Supplementary material associated with this article can be found, in the online version, at doi:10.1016/j.addicn.2025.100233.

## Data Availability

We have shared our data repository in the paper.
