# Supplementary material for: Recruitment of specific dopamine neuron sub-circuits by opioids
Source: Addict Neurosci. Author manuscript; Available in PMC 2026 Jan 6. (PMC12768494; doi:10.1016/j.addicn.2025.100233)
Supplement: 2 [file NIHMS2131412-supplement-2.docx]

Supplemental Figure Legends

Supplemental Figure 1: Immediate cFos staining of morphine treated animals shows TH+ neuron recruitment in the PAG and DR. Both PAG (top) and DR (bottom) showed DA neuron recruitment in both saline (left) and morphine (conditions). Significance not reached in either case (p=0.2410, both).

Supplemental Figure 2: Transgenic cFos capture of morphine treated animals shows minimal TH+ neuron recruitment in the PAG and DR. Both PAG (top) and DR (bottom) showed tDT+ capture of DA neurons in both saline (left) and morphine (right) conditions. Morphine counts were not significantly above saline in either region (PAG p=0.1491, DR p=0.2333)

Supplemental Figure 3: Transgenic capture of fentanyl treated animals shows minimal TH+ neuron recruitment in the PAG and DR. Both PAG (top) and DR (bottom) show tDT+ neuron capture in glycerol (left) and fentanyl (right) conditions. Fentanyl and glycerol counts were not significantly different in either region (PAG p=0.8242, DR p=0.2857) (Fig. 3c).

Supplemental Figure 4: Morphine and fentanyl effects were not significantly different across all regions. Between morphine and fentanyl (left), the cell counts differences within region were not significantly different (Mann U Whitney, p threshold 0.05). Between saline and glycerol (right), the cell counts differences within region were not significantly different (Mann U Whitney, p threshold 0.05, p ≥ 0.0558 all). Across all sections, the effect of morphine (relative to saline) was roughly 0.806 times (95% CI 0.005-1.607) that of fentanyl (relative to glycerol).

Supplemental Figure 5: Projection capture in the fentanyl condition mimics the projection pattern captured in the morphine condition. In sections 43 (a), 44 (b), 45 (c), and 49 (d), fentanyl-captured projections qualitatively recapitulate the dmsACB bias over other ACB nuclei (a-c) and BST enrichment (d).

Supplemental Figure 6: Projections observed to the PFC and amygdalar nuclei. a) Projections to the PFC (bregma +1.845mm, section 36) and b) BLA (bregma -2.055, section 75) were observed consistently, bilaterally, and across morphine over saline brains. The sparseness of these projections decreased efficacy of density calculations to accurately represent the pattern. CEA may show an increase, however, this region was excluded from analysis due to presence of background labeling in some soma.

Supplemental Figure 7: Quantification of intensity in BST and other regions in section 49 reveals no difference compared to controls. a. Across the striatum, BST, and ACB at section level 49, despite visible projections in the BST region, b. none reached statistically significant differences between morphine and saline condition (p ≥ 0.05 all)

Supplemental Figure 8: Aldh1a1 marks a portion of captured TH+ cells in timed cFos staining. a) Rostrocaudal sections comparing cFos+ and Aldh1a1+ cells b) (top) all Aldh1a+ cells were also cFos+ TH+, proportion was calculated per animal (mean, standard deviation) and (bottom) counts of Aldh1a1+ cFos+ TH+ costaining neurons per animal (mean, standard deviation)

Supplemental Figure 9: Behavioral tracking for animals in the fentanyl paradigm. Left: Nosepoke responses for animals in the fentanyl paradigm (see Figure 3a) showing active nosepoke (AN) over total nosepokes for the self-administration portion of the paradigm, days 1-5, mean and standard deviation. Right: number of fentanyl reward deliveries (mean and standard deviation) on each day (1-5) during self-administration, and the passive deliveries on day 5 (which were equivalent at 30 for all subjects).

Supplemental Figure 10: Proportion of Calb1+ DA neurons of the SN and VTA. Left: total count values and right: proportional measures of Calb1+ DA neurons captured in the VTA and SN in the morphine/saline paradigm (see Figure 2a). Calb1 co-labeled neurons comprised 94.8% and 93.3% of captured cells in the VTA and SN, respectively, in the morphine condition. In the saline condition, Calb1 co-labeled cells comprised 81.4% and 100% of captured cells in the VTA and SN, respectively. Note, in the SN most captured neurons are likely to be in the SN dorsal tier and in the SNl. However, since some Aldh1a1+ neurons were also captured (Fig. 5), it is plausible that there may be a few DA neurons that co-express *Aldh1a1* and *Calb1*. Alternatively, limited Aldh1a1+/Calb1+ neurons located at the junction of VTA and SNc may have been counted as SNc due to the imprecise anatomical boundaries of these structures.

Supplemental Table 1: Estimates of F-M differences in soma counts in the immediate cFos staining condition. Using mixed-effects negative binomial model, we examined estimate of contrast between females and males per brain region and condition. These estimates are too imprecise to draw a conclusion about sex differences.
